# Effect of vegetable oils applied over acquired enamel pellicle on initial erosion

**DOI:** 10.1590/1678-7757-2016-0436

**Published:** 2017

**Authors:** Franciny Querobim IONTA, Catarina Ribeiro Barros de ALENCAR, Poliana Pacifico VAL, Ana Paula BOTEON, Maisa Camillo JORDÃO, Heitor Marques HONÓRIO, Marília Afonso Rabelo BUZALAF, Daniela RIOS

**Affiliations:** 1 Universidade de São Paulo, Faculdade de Odontologia de Bauru, Departamento de Odontopediatria, Ortodontia e Saúde Coletiva, Bauru, SP, Brasil.; 2 Universidade Estadual da Paraíba, Faculdade de Odontologia, Centro de Ciência e Tecnologia em Saúde, Campina Grande, PB, Brasil.; 3 Universidade de São Paulo, Faculdade de Odontologia de Bauru, Departamento de Dentística, Endodontia e Materiais Odontológicos, Bauru, SP, Brasil.; 4 Universidade de São Paulo, Faculdade de Odontologia de Bauru, Departamento de Ciências Biológicas, Bauru, SP, Brasil.

**Keywords:** Tooth erosion, Plant oils, Primary prevention, Dental enamel

## Abstract

**Objective:**

The prevalence of dental erosion has been recently increasing, requiring new preventive and therapeutic approaches. Vegetable oils have been studied in preventive dentistry because they come from a natural, edible, low-cost, and worldwide accessible source. This study aimed to evaluate the protective effect of different vegetable oils, applied in two concentrations, on initial enamel erosion.

**Material and Methods:**

Initially, the acquired pellicle was formed *in situ* for 2 hours. Subsequently, the enamel blocks were treated *in vitro* according to the study group (n=12/*per* group): GP5 and GP100 – 5% and pure palm oil, respectively; GC5 and GC100 – 5% and pure coconut oil; GSa5 and GSa100 – 5% and pure safflower oil; GSu5 and GSu100 – 5% and pure sunflower oil; GO5 and GO100 – 5% and pure olive oil; CON− – Deionized Water (negative control) and CON+ – Commercial Mouthwash (Elmex^®^ Erosion Protection Dental Rinse, GABA/positive control). Then, the enamel blocks were immersed in artificial saliva for 2 minutes and subjected to short-term acid exposure in 0.5% citric acid, pH 2.4, for 30 seconds, to promote enamel surface softening. The response variable was the percentage of surface hardness loss [((SHi - SHf) / SHf )×100]. Data were analyzed by one-way ANOVA and Tukey’s test (p<0.05).

**Results:**

Enamel blocks of GP100 presented similar hardness loss to GSu100 (p>0.05) and less than the other groups (p<0.05). There was no difference between GP5, GC5, GC100, GSa5, GSu100, GSa100, GSu5, GO5, GO100, CON− and CON+.

**Conclusion:**

Palm oil seems to be a promising alternative for preventing enamel erosion. However, further studies are necessary to evaluate a long-term erosive cycling.

## Introduction

The prevalence of dental erosion has been increasing in recent years^17^. Dental erosion is defined as a chemical process that involves gradual loss of dental hard tissue by intrinsic or extrinsic acids of non-bacterial origin^22^. Advanced stages of this condition may impair esthetics and function, affecting the patient’s quality of life^16^. Therefore, establishing effective preventive and therapeutic approaches focused on the etiopathogenesis of the lesion is required. Preventive measures should start as early as possible and involve causal measures, such as dietary advice, to reduce the erosive challenges. In addition, the development of strategies to enhance biological protective factors may help preventing dental erosion. Saliva has been considered the most important biological factor on the pathogenesis of dental erosion^3,14^. The protective mechanism of saliva includes the formation of the acquired enamel pellicle (AEP)^9,25^, a non-bacterial organic film formed over the enamel surface by the adsorption of proteins, peptides, lipids, and other macromolecules available in saliva^6,9^. The AEP plays an important role on the prevention of dental erosion, working as a mediator that diminishes the direct contact of acids with the enamel surface^9^. The protective potential of the AEP depends on its physical properties, including thickness and maturation time^9^. Studies have shown that pellicles formed during two hours or less offer maximum protection against erosive demineralization, without any increase in enamel erosion prevention with longer periods of maturation^10,26^. One possible strategy to increase AEP protection may be the modification of its composition, to improve the protective effect during an erosive challenge by the maintenance of the AEP on the enamel. Lipids consist of about 25% of the dry weight of acquired pellicle^10^, and it is known that lipophilic components are able to modulate the composition and ultrastructure of the AEP^12^. Therefore, it is believed that lipid-rich AEPs are more resistant to acid challenges, protecting against enamel erosion^12^.

The preventive potential of vegetable oils has been widely studied, since they are a natural, edible, low-cost, and worldwide accessible source^2,8,12^. A previous study showed that 2% olive oil and 2% olive oil associated to fluoride mouthwash were able to prevent erosion, but to a lower extent when compared with the positive control (acidulated fluoride solution, 250 ppm, pH 3.88)^27^. Various types of vegetable oils are available and their anti-erosive potential might be different according to their composition, including the types of fatty acids and other components. This study aimed to evaluate the *in vitro* effect of different types of vegetable oils, in pure form or as emulsions, applied on AEP formed *in situ*, on the protection of enamel against initial erosive demineralization.

## Material and methods

### Experimental design

This study was conducted according to the Declaration of Helsinki. The protocol was approved by the local Research Ethics Committee (Protocol 1.173.522/2015). All individuals signed an informed consent form before the confirmation of their eligibility for the study.

**Figure 1:**
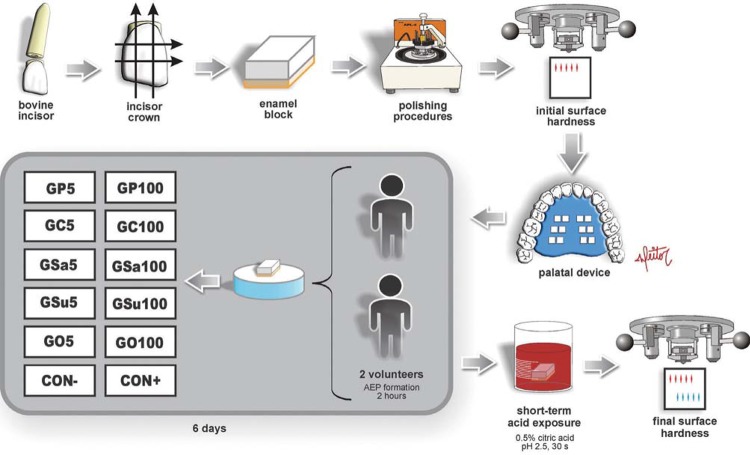
Illustration of the experimental design adopted in this study

This study evaluated the *in vitro* potential of distinct vegetable oils, in different concentrations, to inhibit enamel erosive demineralization. The experimental design is shown in Figure 1. Before applying the oils, the AEP was formed *in situ* on 24 enamel blocks worn by two volunteers (1 block for each group per volunteer) for 2 hours. Subsequently, the enamel blocks were treated *in vitro* according to the groups (n=12/per group): GP5 – 5% palm oil; GP100 – pure palm oil; GC5 – 5% coconut oil; GC100 – pure coconut oil; GSa5 – 5% safflower oil; GSa100 – pure safflower oil; GSu5 – 5% sunflower oil; GSu100 – pure sunflower oil; GO5 – 5% olive oil; GO100 – pure olive oil; Control− – negative control, deionized water; Control + – positive control, mouthwash commercial solution containing 125 ppm F^−^ as AmF, 375 ppm F^−^ as NaF, 800 ppm Sn^2+^ as SnCl_2_; pH 4.5 (Elmex^®^ Erosion Protection Dental Rinse/EP – CP GABA GmbH; Hamburg, Germany). After application of the oils (5 drops, 30 seconds), the blocks were immersed in 0.5% citric acid (nascent pH 2.4) during 30 seconds to promote the softening of enamel surface. The percentage of surface hardness change was assessed (response variable). The mentioned procedures were repeated for 6 days, in which one sample *per* group was fixed in each volunteer intraoral appliance *per* day.

### Sample size

A pilot study was conducted with six enamel blocks of 100% palm oil and negative control (deionized water) *per* group. Thus, a standard deviation of 8.5% was obtained. Twelve samples per group were set, considering 12 groups with a minimally detectable difference of 15% in hardness loss and 8.5% of standard deviation, with alpha and beta errors of 5% and 20%, respectively.

### Enamel blocks preparation

Enamel blocks (4×4×3 mm_3_, n=160) were prepared from the labial surfaces of bovine incisor crowns. The blocks were cut using a IsoMet^®^ low speed saw cutting machine (Buehler Ltd.; Lake Bluff, Illinois, United States) with two diamond disks (Extec Corp.; Enfield, Connecticut, United States), which were separated by a 4-mm thickness spacer. The blocks’ surfaces were smoothed with water-cooled silicon carbide discs (320, 600, and 1200 grade papers; Buehler Ltd.; Lake Bluff, Illinois, United States), and wet polished with felt paper and diamond spray (1 µm; Buehler Ltd.; Lake Bluff, Illinois, United States). The blocks were cleaned using an ultrasonic device for 2 min and verified regarding the presence of white spots and cracks using a microscope (40×). Knoop surface hardness (SH*i*) was determined by the mean values of five indentations performed 100 µm away from each other, with 25 g for 10 seconds (Micromet^®^ 5114 hardness tester; Buehler Ltd., Lake Bluff, Illinois, United States). One hundred and forty four enamel blocks were selected by excluding values 10% higher or lower than the mean microhardness of all specimens (interblock variability), to avoid bias regarding initial enamel condition. The blocks were allocated using Microsoft Excel to distribute blocks with lower and higher initial hardness values equally into all groups. The randomization was done to divide the enamel blocks between the groups and the two volunteers (position of the block in the intraoral device and 6 days of experiment).

Before the *in situ* phase for AEP, the blocks were sterilized with ethylene oxide^23^.

### *In situ* phase – acquired enamel pellicle formation

Two healthy adult volunteers with the same age (22 years old), residing in the same fluoridated area (0.70 mg F/l), participated in the study, after satisfying the following inclusion criteria: physiological salivary parameters (stimulated >1 ml/min; unstimulated >0.1 ml/min; neutral pH 7.0-7.5); absence of erosive tooth wear, untreated carious lesions, or periodontitis. The exclusion criteria were: presence of systemic diseases; use of medicines that affect the salivary characteristics (antidepressants, narcotics, diuretics, or antihistamines); undergoing radiation or chemotherapy; gastro-esophageal reflux; frequent regurgitation and/or vomiting; pregnancy or breastfeeding; smoking; practicing pool activities (exposure to low-pH treated water); working in low-pH environment (e.g., batteries industry); or fluoride topical application in the past two months. The intraoral palatal appliances were made with acrylic resin containing six sites (9×6×3 mm) for two blocks fixation in each (n=12).

The position of the blocks in the intraoral appliance was randomly determined for each volunteer. Seven days prior to and during the experiment, the volunteers brushed their teeth with commercial fluoride toothpaste containing 1,450 ppm F (Tripla Ação^®^ – Colgate-Palmolive Comercial Ltda; São Paulo, São Paulo, Brazil). The volunteers were also warned not to use any other fluoride product. Toothbrushing with fluoride toothpaste was performed by the volunteers one hour prior to the insertion of the intraoral appliance. During 6 days, at the same time (8-10 AM), two volunteers used the intraoral appliances containing one block for each studied group during 2 hours to allow the formation of the AEP. The volunteers did not eat or drink in this period.

### *In vitro* phase – treatment and acid exposure

Immediately after the formation of the AEP, the enamel blocks were removed from the intraoral appliance and fixed in an acrylic disk to receive the treatment. The commercial brands of the vegetable oils used in this study were: GP5 and GP100: palm oil (Kidendê - Dendê Light Indústria de Produtos Alimentícios Ltda; Valença, Bahia, Brazil); GC5 and GC100: extra virgin coconut oil (COPRA - COPRA Indústria Alimentícia Ltda; Maceió, Alagoas, Brazil); GSa5 and GSa100: extra virgin safflower oil (Giroil - Giroil Agroindústria Ltda, Entre-Ijuís, Rio Grande do Sul, Brazil); GSu5 and GSu100: extra virgin sunflower oil (Pazze - Pazze Indústria de Alimentos Ltda; Panambi, Rio Grande do Sul, Brazil); GO5 and GO100: extra virgin olive oil (Cirio - Sandeleh Alimentos; Paranaguá, Paraná, Brazil).

The 5% emulsions of the vegetable oils in deionized water were daily prepared prior to the application by using a high-speed household mixer, resulting in a finely dispersed emulsion^27^.

The treatment consisted in applying five drops on each enamel block of the respective group during 30 seconds. Then, the enamel block was separately immersed in 17.6 ml of artificial saliva (0.33 g KH_2_PO_4_, 0.34 g Na_2_HPO_4_, 1.27 g KCl, 0.16 g NaSCN, 0.58 g NaCl, 0.17 g CaCl_2_, 0.16 g NH_4_Cl, 0.2 g urea, 0.03 g glucose, 0.002 g ascorbic acid, pH 7^13^ – without mucin) for 2 minutes, under constant agitation, to simulate a natural rinsing process occurring in the oral cavity. After that, the enamel blocks were subjected to short-term erosive demineralization by immersing each block in 17.6 ml of 0.5% citric acid pH 2.4, under constant agitation, for 30 seconds. Then, the blocks were washed with deionized water for 30 seconds.

### Surface hardness assessment

After the short-term acid exposure, the surface hardness determination was performed again (SH*f*) with five indentations performed at 100 µm distance in relation to initial indentations (Micromet^®^ 5114 hardness tester; Buehler Ltd., Lake Bluff, Illinois, United States). The percentage of hardness loss was calculated [((SH*i* - SH*f*) / (SH*i*)) × 100] for each block and averaged to represent the studied groups.

### Statistical analysis

Statistical analysis was performed with SigmaPlot™ version 12.3 (Systat Software GmbH; Erkrath, Germany). Assumptions of equality of variances and normal distribution of errors were verified by Bartlett’s and Shapiro–Wilk tests, respectively. Once the assumptions were satisfied, two-way ANOVA (for the factors “volunteers” on two levels and “treatments” on 12 levels) and Tukey’s *post hoc* test were applied. The significance level was set at 5%.

## Results

We found no statistically significant difference for the factor “volunteers” (p=0.911), and no interaction between “volunteers” and “treatments” (p=0.634), but we found significant difference between “treatments” (p=0.002). The percentage of hardness loss of the evaluated groups is shown in Table 1. Only GP100 (pure palm oil) was statistically different from the control group, showing the lowest surface hardness loss (p<0.05). All the other studied oils presented surface hardness loss similar to the control groups (p>0.05). We found no significant difference between GP100 and GSu100 (pure sunflower oil) (p>0.05).

**Table 1:** Mean and standard deviation (±SD) of the percentage of hardness loss of enamel treated with the studied vegetable oils

Groups	SHi	SHf	% Hardness Loss
GP5 – 5% palm oil	329.92 (±35.81)	253.90 (±45.15)	23.24 (±8.436)^a^
GP100 – pure palm oil	337.58 (±28.41)	310.80 (±34.58)	7.89 (±7.5)^b^
GC5 – 5% coconut oil	334.16 (±26.88)	250.92 (±37.49)	24.65 (±11.50)^a^
GC100 – pure coconut oil	336.24 (±30.50)	240.26 (±48.45)	28.47 (±13.37)^a^
GSa5 – 5% safflower oil	341.19 (±31.87)	241.90 (±37.23)	28.74 (±11.53)^a^
GSa100 – pure safflower oil	337.85 (±29.91)	248.89 (±43.01)	26.56 (±9.51)^a^
GSu5 – 5% sunflower oil	335.76 (±26.05)	259.23 (±50.07)	22.92 (±12.94)^a^
GSu100 – pure sunflower oil	338.51 (±27.63)	265.02 (±55.67)	21.78 (±14.83)^ab^
GO5 – 5% olive oil	337.27(±32.29)	252.81 (±57.71)	25.35 (±12.76)^a^
GO100 – pure olive oil	337.36 (±29.49)	249.86 (±46.95)	25.91(±12.51)^a^
CON− – deionized water (negative control)	335.45 (±29.71)	240.90 (±38.02)	28.09 (±9.95)^a^
CON+ – fluoride mouthrinse (positive control)	337.19 (±28.92)	256.44 (±22.04)	23.74 (±6.15)^a^

In the fourth column, different letters show significant differences between the groups (two-way ANOVA and Tukey’s test, p<0.05)

## Discussion

Lipids consist of about 25% of the pellicle’s dry weight^19^, and lipophilic components are able to modulate the pellicle composition and ultrastructure^12^. Therefore, authors have suggested that lipid-rich pellicles might be more resistant to acids^23^, thus reducing erosion^12^.

This study aimed to elucidate the protective effect of different vegetable oils, applied after *in situ* formation of AEP, against initial erosion demineralization. Two of the vegetable oils assessed here (coconut oil and palm oil) have not been previously studied regarding their anti-erosive potential, requiring an initial *in vitro* evaluation of their effect. However, *in vitro* studies are not able to accurately replicate the biological characteristics of the oral cavity, such as the presence of human saliva and the formation of AEP, which could interfere with the development of erosion. Some limitations occur in protocols using human saliva *in vitro*, such as fast extraoral protein decomposition^18^. Natural and *in vitro* formed AEPs also present differences in their characteristics, e.g., the natural pellicle is more hydrophobic than the one formed *in vitro* ^24^. Therefore, a combined *in situ*/*in vitro* protocol was chosen in this study to allow the physiological formation of the AEP *in situ* prior to the *in vitro* application of the vegetable oils and short-term exposure to citric acid. A single short-term erosive challenge was performed to more precisely evaluate the protective ability of the AEP modified by the studied oils against initial enamel erosion. Studies have shown that the hardness test is an adequate method to evaluate the initial softening of enamel surface^11,20^.

Vegetable oils are extracted from oil plants and seeds and are commonly used in foods, cosmetics, and medical products^12^. Studies have shown that, when hard tooth tissue is exposed to vegetable oils, the superficial layer of the AEP gets rich in lipids micelles^4,7^. However, the protective effect of these oils against caries and erosion demineralization processes remains unclear, because only a few evidence-based researches are available in the literature^2,8,27^.

Only one study evaluated the direct effect of 5 and 50% olive oil emulsions compared to distilled water and fluoride solution on dentin caries demineralization^2^. Dentin samples were subjected to three cycles *per* day of 5 min treatment application followed by 8 hours of immersion in demineralization solution (pH 5.0) during 9 days. The olive oil emulsions showed a decrease in mineral loss in comparison with deionized water, and the fluoride solution presented better results^2^.

Our results showed that pure palm oil was capable to protect enamel against initial erosion demineralization, but the same was not found for the 5% palm oil emulsion. No protective effect was observed to 5% emulsion and pure form of coconut, safflower, sunflower, and olive oils. The effect of olive oil-based emulsions (100%, 2%, and 2% associated with mouthwash) on enamel subjected to 10 erosive cycles was previously assessed using profilometry analysis^27^. Each cycle consisted of samples treatment with oil-based emulsions during 5 min, immersion in artificial saliva for 30 min, demineralization in 1% citric acid for 3 min, and remineralization in artificial saliva for 60 min. The researchers found that 2% emulsion or 2% olive-oil containing mouthrinse offered protection against tooth erosion, but in a lesser extent than the positive control (250 ppm acidic fluoride solution), and that pure olive oil did not offer protection^27^. The authors hypothesized that the adhesion properties of olive oil might increase when applied as emulsion^27^. In contrast, our study did not find any protective potential for 5% and pure olive oil. The different results between the studies might be explained by the different methodologies used. We adopted a short-term erosive demineralization model and the abovementioned study used an erosive cycling model.

The effect of safflower oil on the protective properties of the AEP formed *in situ* against the exposure to hydrochloric acid for 2 min was previously described^8^. Enamel mineral loss was determined by measurement of calcium and phosphate release, and the ultrastructure of the AEP was evaluated by transmission electron microscopy. The results showed that the surface of AEP was rich in lipids, but no substantial lipids integration was found in the pellicle’s basal layer. The *in situ* AEP modified by safflower oil rinsing was more susceptible to acid degradation than the *in situ* physiological AEP^8^. In contrast, our study showed that safflower oil (GSa5 and GSa100) did not present a negative impact on enamel demineralization when compared to the control groups.

To our knowledge, palm oil has never been investigated for the prevention of erosion. Palm oil is the second largest produced and consumed vegetable oil in the world, due to its high productivity, low production cost, and rich nutritional content^21^. It is rich in tocotrienols that have presented health benefits^1^. Tocotrienols allow an efficient penetration into tissues that have saturated fatty layers and exhibit antioxidant protection of cellular membranes against oxidative damage^1^. The antioxidant property has been attributed to its ability of distribution in lipid layers of the cell membrane^1^.

In previous studies, the outer layer of the AEP was modified by the increase of lipids micelles^4,7^. However, the outer layers of the AEP are supposed to be easily removed after an erosive challenge, in contrast to the basal layer that might not be affected^6^. In this study, despite the ultrastructure of the AEP not being analyzed, it is hypothesized that palm oil might have modified the basal layer of the acquired pellicle, increasing its protective potential. The tocotrienols contained in the palm oil might have allowed its penetration and distribution into the basal layers of the acquired pellicle, increasing its protective role^1^. We also highlight that we found no differences between the protective effect of pure palm oil and pure sunflower oil. This result can be explained by the tocotrienols content of the sunflower oil, but in a lesser extent when compared to palm oil^1^, which enables a borderline behavior between palm oil and the other tested oils (coconut, safflower, and olive oil).

In this study, the commercial mouthwash solution – Elmex^®^ Erosion Protection Dental Rinse/EP, 125 ppm F^−^ as AmF, 375 ppm F^−^ as NaF, 800 ppm Sn^2+^ as SnCl_2_; pH 4.5 (CP GABA GmbH; Hamburg, Germany) – did not present any effect on the inhibition of initial enamel erosion, being similar to deionized water (negative control). The role of fluoride on tooth erosion is not fully evidenced^14^. Differing from our result, some studies have shown a preventive capacity of fluoride solution containing AmF/NaF (500 ppm F) and SnCl_2_ (800 ppm Sn) against enamel erosion^5,15^.

Although palm oil has shown superior protective capacity against tooth erosion, its effect to prevent the enamel erosive wear needs to be further evaluated under long-term erosive challenges. Moreover, the effect of palm oil on the physical properties, quality, and composition of the acquired pellicle should also be assessed.

## Conclusion

Considering our study design, palm oil seems to be a promising alternative for the prevention of initial enamel erosion.
